# Genetic structure in Red Junglefowl (*Gallus gallus*) populations: Strong spatial patterns in the wild ancestors of domestic chickens in a core distribution range

**DOI:** 10.1002/ece3.4139

**Published:** 2018-06-11

**Authors:** Hoa Nguyen‐Phuc, Mark E. Berres

**Affiliations:** ^1^ Department of Animal Sciences University of Wisconsin‐Madison Madison Wisconsin; ^2^ Department of Ecology and Evolutionary Biology Vietnam National University HCMC Ho Chi Minh City Vietnam; ^3^ Biotechnology Center University of Wisconsin‐Madison Madison Wisconsin

**Keywords:** AFLP, Bayesian cluster analysis, dispersal, gene flow, population structure, Red Junglefowl

## Abstract

Red Junglefowl (*Gallus gallus*) are among the few remaining ancestors of an extant domesticated livestock species, the domestic chicken, that still occur in the wild. Little is known about genetic diversity, population structure, and demography of wild Red Junglefowl in their natural habitats. Extinction threats from habitat loss or genetic alteration from domestic introgression exacerbate further the conservation status of this progenitor species. In a previous study, we reported extraordinary adaptive genetic variation in the MHC B‐locus in wild Red Junglefowl and no evidence of allelic introgression between wild and domestic chickens was observed. In this study, we characterized spatial genetic variation and population structure in naturally occurring populations of Red Junglefowl in their core distribution range in South Central Vietnam. A sample of 212 Red Junglefowl was obtained from geographically and ecologically diverse habitats across an area of 250 × 350 km. We used amplified fragment‐length polymorphism markers obtained from 431 loci to determine whether genetic diversity and population structure varies. We found that Red Junglefowl are widely distributed but form small and isolated populations. Strong spatial genetic patterns occur at both local and regional scales. At local scale, population stratification can be identified to approximately 5 km. At regional scale, we identified distinct populations of Red Junglefowl in the southern lowlands, northern highlands, and eastern coastal portions of the study area. Both local and long‐distance genetic patterns observed in wild Red Junglefowl may reflect the species’ ground‐dwelling and territorial characteristics, including dispersal barriers imposed by the Annamite Mountain Range. Spatially explicit analyses with neutral genetic markers can be highly informative and here elevates the conservation profile of the wild ancestors of domesticated chickens.

## 1 INTRODUCTION

Domestication of wild animals is considered one of the major milestones in human civilization (Diamond, 2002). Along the course of human history, approximately 40 livestock species have been domesticized, many of which contribute substantially to modern agricultural and food production (FAO, 2007). Domestication of wild animals is considered a complex and cumulative process of gradually altering behaviors and morphological characteristics of wild animals to be compatible with human stewardship (Diamond, 2002). Historically, the process was believed to have taken place in different parts of the world (Bruford, Bradley, & Luikart, 2003; Lenstra et al., 2012). Despite the involvement of many progenitor stocks (e.g., wild species) or lines, only a limited number of domesticated animal species exist today. The cumulative effect of artificial selection for domestication not only differentially shaped the target animals to produce favorable characteristics and behaviors for humans but apparently has also impacted their wild progenitors. With the exception of the Wild Boar (*Sus scrofa*) and Red Junglefowl (*Gallus gallus*), the ancestors and wild relatives of existing livestock are considered extinct or highly endangered in their natural habitats (Bruford et al., 2003). The largely vanished diversity of domestic animal ancestors emphasizes the importance of the few remaining progenitor lines: They can help us understand the history of domestication, the evolution of domesticated animals, and may even represent an important source of genetic variation and adaptive traits for future agriculturally focused breeding programs.

In these respects, Red Junglefowl are an important species to study. Red Junglefowl still commonly exist in their native habitats (Brickle et al., 2008; Brisbin, 1995) and are clearly distinguishable from domestic chickens (Johnsgard, 1999). Although genetic contributions from multiple Junglefowl species may have played a role in the domestication process (Eriksson et al., 2008; Nishibori, Shimogiri, Hayashi, & Yasue, 2005), archeological and genetic evidence (Fumihito et al., 1994, 1996; Gongora et al., 2008; Storey et al., 2012; Thomson et al., 2014) indicate that Red Junglefowl from Southeast Asia was the primary progenitor of all domestic breeds of modern chickens. In a previous study, we identified substantial haplotype variation in the major histocompatibility complex (MHC) B‐locus of wild Red Junglefowl (Fulton et al., 2016; Nguyen‐Phuc, Fulton, & Berres, 2016). Curiously, none of the Red Junglefowl haplotypes were found in a large sample of commercial and heritage chicken breeds (Fulton et al., 2016; Nguyen‐Phuc et al., 2016). This result has many open interpretations including that Red Junglefowl in Vietnam may not be the direct ancestor of domestic chickens or that the MHC B‐locus in domestic chickens was altered radically by artificial selection.

To trace ultimately the ancestral role and contributions of Red Junglefowl in chicken domestication, it is necessary to emphasize the importance of ecology, behavior, and distribution of *wild* Red Junglefowl. Jaap and Hollander (1954) first proposed that the Red Junglefowl be the standard genetic wild type of chickens. Brisbin and others further described the wild‐type ecology and morphologies of Red Junglefowl in their natural habitats (Brisbin, 1995; Brisbin & Peterson, 2007; Brisbin, Peterson, Okimoto, & Amato, 2002; Peterson & Brisbin, 1998). These works emphasized the implications of captive Red Junglefowl possibly being combined with genes of domestic origin (Peterson & Brisbin, 1998) and the necessity to sample wild‐type Red Junglefowl in their natural habitats in the future genetics studies (Brisbin et al., 2002).

Most, if not all, recent genetic studies involving junglefowl, however, sample birds from captive colonies (e.g., Berthouly et al., 2009; Eriksson et al., 2008; Fumihito et al., 1994; Gering, Johnsson, Willis, Getty, & Wright, 2015; Mekchay et al., 2014; Moiseyeva, Romanov, Nikiforov, Sevastyanova, & Semyenova, 2003; Romanov & Weigend, 2001; Rubin et al., 2010; Tadano et al., 2008; Worley et al., 2010) or from vaguely described geographic localities (e.g., Akaboot, Duangjinda, Phasuk, Kaenchan, & Chinchiyanond, 2012; Granevitze et al., 2007; Liu et al., 2006; Miao et al., 2013; Nishibori et al., 2005; Okumura et al., 2006; Ulfah et al., 2016). Even the female Red Junglefowl individual used for the *Gallus gallus* reference sequence (International Chicken Genome Sequencing Consortium, 2004) is traceable to the San Diego Zoo, itself believed to be introgressed with White Leghorn alleles (M. E. Delany, University of California, Davis, CA, personal communication).

Therefore, the general use of the name Junglefowl (e.g., Red Junglefowl) unfortunately implies the “wild” condition but in fact usually refers to captive‐bred individuals. One explanation posits that wild Junglefowl individuals—including adult males, females, juvenile wild chicks, or wild chicks hatched by domestic chickens—do not tolerate captivity (personal observations, Brisbin et al., 2002; Codon, 2012; Collias & Collias, 1996). Thus, Red Junglefowl obtained from captive populations must have been crossed with domestic breeds in order to maintain them (Brisbin, 1995). Indeed, only after three or four generations of crossing wild male Red Junglefowl to female domestic chickens (e.g., small heritage breeds such as Vietnamese “ga tre”) will offspring survive sufficiently well and eventually tolerate a continued human presence (H. Nguyen‐Phuc and M. E. Berres, unpublished data). Efforts to maintain wild‐type Red Junglefowl have been attempted but usually end in failure, possibly due to genetic incompatibilities and/or specific nutrient deficiencies affecting early hatchings (Brisbin et al., 2002; Nguyen‐Phuc and Berres, unpublished data).

Our current study aimed to characterize spatial genetic diversity and population structure of wild Red Junglefowl in their natural habitats and with a landscape context. The major research objective was to investigate how spatial processes of landscape and ecology influence genetic characteristics of Red Junglefowl in their core distribution range. We employed a landscape genetics research framework (Manel, Schwartz, Luikart, & Taberlet, 2003) to test the hypothesis that Red Junglefowl exhibit inter‐ and intra‐population variation across the landscape due to physical distance, dispersal barriers, and species‐intrinsic demographic characteristics. We demonstrate the utility of distance‐ and model‐based Bayesian clustering at different spatial resolutions to investigate this remarkable and important species of pheasant.

## 2 METHODS

### 2.1 Field sampling

**Figure 1:**
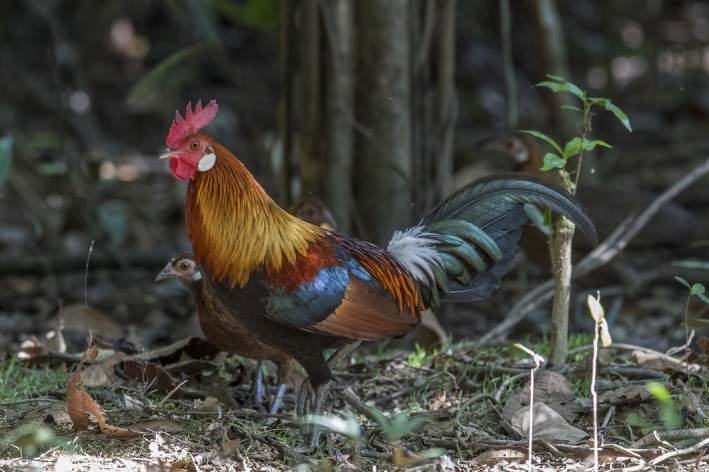
Territorial Red Junglefowl male (front) in breeding plumage with several females (rear), Cat Tien National Park, Vietnam

**Figure 2:**
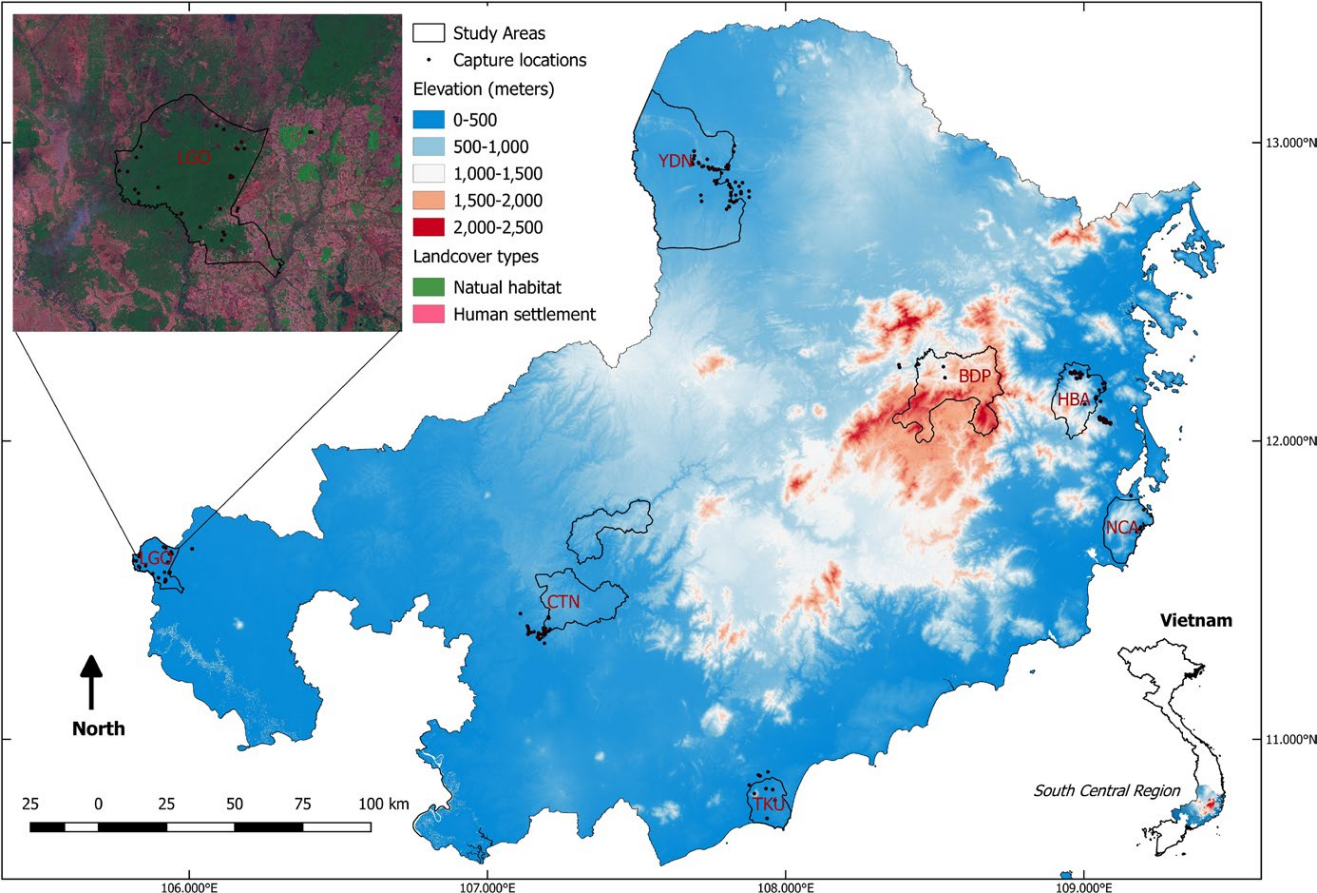
Sampling sites with the Annamite Mountain Range elevation. Bi Doup—Nui Ba National Park (BDP), Cat Tien National Park and Dong Nai Nature Reserve (CTN), Hon Ba Nature Reserve (HBA), Lo Go Sa Mat National Park (LGO), Nui Chua National Park (NCA), Ta Kou Nature Reserve (TKU), and Yok Don National Park (YDN). Insert (LGO) presents example of isolated natural habitats in South Central Vietnam

We sampled Red Junglefowl (Figure 1) in seven protected areas in South Central Vietnam (Figure 2). South Central Vietnam is the core distribution range of Red Junglefowl (Johnsgard, 1999) and within the larger range of the species in the Indo‐Burma biodiversity hotspot (Brickle et al., 2008). Major biogeographical and climatic features of South Central Vietnam are characterized by the Annamite Mountain Range (Truong Son). It is comprised of extended mountains and high plateaus covering mountainous areas ranging in elevation from 600 to 2,400 m and approximately 200 km from east to west and 1,100 km from north to south (Sterling & Hurley, 2005). Within the Annamite Range, South Central Vietnam has diverse landscapes and different micro‐climatic zones, which may modulate the distribution of Red Junglefowl in the region. Tropical lowland forests occur in the eastern coastal areas in South Central Vietnam and become more abundant in the south. Seasonally dry, deciduous forests are found mainly in the northwest and coniferous forests dominate higher elevations. Historically, Red Junglefowl likely were continuously distributed over the entirety of Vietnam with the Annamite Range acting possibly as an extended physical dispersal barrier. The current landscape throughout much of South Central Vietnam is comprised of small patches of severely fragmented natural habitats (Figure 2 insert), some of which are protected as nature reserves or national parks. Our sampling sites included the following: Bidoup Nui Ba National Park (BDP), Cat Tien National Park and Dong Nai Nature Reserve (hereafter CTN as the two sites are connected), Hon Ba Nature Reserve (HBA), Lo Go Sa Mat National Park (LGO), Nui Chua National Park (NCA), Ta Kou Nature Reserve (TKU), and Yok Don National Park (YDN) (Figure 2). These areas consist the majority of protected and natural habitats in South Central Vietnam. Selection of these sites was also based on the presence of suitable Red Junglefowl habitat and a relatively symmetrical distance from the Annamite Mountain Range. The average area of each field site was approximately 50,000 ha. These protected areas feature mostly undisturbed habitats containing lowland tropical rainforest (≤600 m in elevation) and seasonally dry deciduous forest. The sampling sites are separated from each other by approximately 120 km, interspersed with urban and non‐natural areas such as farms (Figure 2 insert).

We live‐captured Red Junglefowl during three dry seasons in 2012, 2013, and 2014 with nonlethal walk‐in leg snare traplines (Bub, 1991) adapted to reflect local trapping customs and field conditions. Tropical dry season in the Annamite Mountain Range occurs from January to May and they overlap with the primary breeding season of Red Junglefowl. Mating and territorial defense facilitate the location and sampling of birds. Outside of this period, Red Junglefowl become secretive and extraordinarily difficult to locate and therefore not easily captured. We employed opportunistic sampling depending on the density of territorial Junglefowl and capture opportunities. However, most of our capture sites were in undisturbed habitats and far from human settlements.

Each trapline was approximately 100 m in length and designed to capture territorial Junglefowl. Traplines were made by modifying vegetation at the capture site into a barrier approximately 1 m high *×* 1 m wide to encourage Junglefowl walk into snares. Interspersed along the trapline were 20 to 25 open breaks that contained motion‐triggered nonlethal leg‐capture snares. Each snare had a slip‐knot noose with approximately 10 cm opening at one end and to the other end attached to a spring pole, normally a small bamboo twig. The noose was placed on trigger stick situated about 2–3 cm above the ground. When the noose was tightened and pulled from the Junglefowl’s leg, the trigger stick was pulled thus engaging the trap. The tension only tightened the noose around the bird’s leg, which rendered the bird tethered on the ground. Traps were inspected at 30‐min intervals from approximately dawn to dusk. We also employed domestic roosters trained to produce visual and audile challenges to territorial wild Red Junglefowl males (“baiting cocks”) to augment our trapping efforts.

Each captured Red Junglefowl was marked with a uniquely numbered aluminum band (National Band & Tag Company, KY). At each capture site, we recorded geographical coordinates with a GPS receiver and characterized major vegetation structure. A small blood sample (20–200 μl) was obtained from venipuncture of the brachial vein and stored in a modified lysis buffer (0.1 mol/L Tris–HCl pH 8.0, 0.01 mol/L EDTA, 4% SDS) (Longmire, Maltbie, & Baker, 2000) until nucleic acid purification. We used a higher SDS concentration than the original 2% to better lyse blood cells and preserve DNA in high temperature field conditions. After processing, each bird was released at the site of capture, typically within 5 min of capture.

### 2.2 Generation of AFLP profiles

We generated amplified fragment‐length polymorphism (AFLP) fingerprints (Vos et al., 1995) using modified protocols (Marschalek & Berres, 2014). Genomic DNA was purified from blood using the Promega Wizard DNA Isolation kit (Promega Corp., Madison, WI, USA) and assessed visually for nondegraded, high molecular weight DNA with 1% agarose gel electrophoresis and ethidium bromide staining. We screened various combinations of restriction enzyme pairs, preselective and selective primer pairs, and chose two pre‐ and selective primer pairs that maximized the number of fragments meeting a criterion of electrophoretic size (50–650 bp) and resolution (Berres, 2003). A known value of 200 ng of purified DNA was digested to completion with 20 U *Eco*RI (5′‐G|AATTC‐3′) and 20 U *Ase*I (5′‐AT|TAAT‐3′) at 37°C overnight followed by a 20‐min heat inactivation at 65°C. Digested fragments were ligated to double‐stranded oligonucleotide adapters with overhangs complementary to the digested ends with 400 U T4 DNA ligase (NEB, Ipswich, MA) overnight at 16°C. Ligated fragments were diluted 1:4 with 10 mmol/L Tris–HCl (pH 8.0) to produce DNA templates suitable for polymerase chain reaction (PCR) amplification.

Preselective PCR was performed in 50 μl volumes containing 10 μl diluted ligation mixture with 1× GoTaq Flexi Buffer, 1.5 mmol/L MgCl_2_, 0.05 mmol/L dNTP, 2% deionized formamide, 1.25 U Taq DNA Pol I, and 15 pmoles each of primer pair *Eco*RI+C/*Ase*I+G or *Eco*RI+G/AseI+G. Thermocycling conditions consisted of one cycle of 72°C for 2 min, an initial denature at 94°C for 1 min followed by 25 cycles each of 94°C for 50 s, 56°C anneal for 1 min, and 72°C extension for 2 min. Preselective amplification products were diluted 1:19 with 10 mmol/L Tris–HCl (pH 8.0). Amplifications with lower amplicon concentration (determined by visual inspection on an ethidium bromide stained 1% agarose gel) were diluted 1:9 with 10 mmol/L Tris–HCl (pH 8.0).

Selective PCR amplification was performed in 25 μl volumes containing 5 μl diluted preselective amplification product with 1× GoTaq Flexi Buffer, 2 mmol/L MgCl_2_, 0.2 mmol/L dNTP, 2% deionized formamide, 0.625 U Taq DNA Pol I and 5 pmoles HPLC‐purified primer *Eco*RI+CAT labeled with 6‐carboxyfluorescein (6‐FAM), and 25 pmoles *Ase*I+GA or with 5 pmoles HPLC‐purified primer *Eco*RI+GG labeled with 6‐carboxyfluorescein (6‐FAM), and 25 pmoles *Ase*I+GC. Thermocycling conditions consisted of an initial 94°C denature for 1 min followed by 10 cycles of a one‐min annealing touchdown (1°C decrease each cycle) from 65 to 56°C each with a 72°C extension for 2 min. The selective amplification was completed with 18 cycles of 95°C for 50 s, 56°C for 1 min, and 72°C for 2 min. Selectively amplified PCR products were purified over Superfine Sephadex G75 (Sigma) and stored at −80°C. One microlitre of purified product was combined with 13.5 μl deionized formamide and 0.5 μl Geneflo 625 mobility standard (CHIMERx Molecular Biology Products, Milwaukee, WI, USA) for electrophoresis on an ABI 3730xl DNA Analyzer (Biotechnology Center, UW‐Madison, WI).

Fingerprints of AFLP markers were categorized (binned) by a criterion of size‐homology by a partially automated scoring process procedure. We used *RawGeno* package (Arrigo, Tuszynski, Ehrich, Gerdes, & Alvarez, 2009) in *R* 3.1.2 open‐source environment (R Development Core Team, 2011) to create groups of homologous amplicons based on their electrophoretic mobility (converted to units of base pairs). Maximum bin width was set at 1.2 base pairs and unconstrained for narrower bin sizes. This procedure generated a binary matrix of the presence/absence (1/0) scores for each marker amplicon in the AFLP fingerprint. Trace files of the individual samples were also visually inspected in *DAx* 8.0 (van Mierlo Inc., The Netherlands). On the ABI 3730xl device, replicable generation of AFLP fingerprints yielded amplicons with a relative fluorescence intensity (RFI) between 500 and 15,000. Peaks with RFI beyond this range were eliminated from the analysis. Comparing bins created by *RawGeno* and *Dax* (each has differing binning algorithm) helped to evaluate discrepancies in marker assignment. In cases where bins of markers did not match, visual inspection and manual bin reconstruction were performed to correct any errors in marker assignment.

### 2.3 General data analysis

We employed individual approaches based on band scores (ordination) and population approaches based on allele frequencies (genetic diversity estimates and Bayesian clustering) in our study. At the individual level, we calculated coefficients of similarity (Jaccard, 1908) for Red Junglefowl AFLP fingerprints using the *R*‐package *ecodist* (Goslee & Urban, 2007). This generated a square matrix of genetic (dis)similarities that were further analyzed with spatial and nonspatial unconstrained ordination and correlograms to determine patterns of genetic differentiation at different spatial scales, that is, clusters, clines, and isolation‐by‐distance (IBD) (Wright, 1943). At the population level, we combined standard population genetic summary statistics and Bayesian inferences to estimate allele frequencies, calculate overall and pairwise genetic differentiation *F*_ST_ values, and identify population clusters. The population‐based methods converted the bi‐allelic AFLP markers into expected allele frequencies implicitly assuming both linkage and Hardy‐Weinberg equilibrium (HWE). Applications of individual‐based approaches and population‐based approaches in AFLP data have been reviewed and discussed (e.g., Bonin, Ehrich, & Manel, 2007; Guillot, Leblois, Coulon, & Frantz, 2009), and the approaches are intrinsically different in their assumptions, complexities, and computational requirements. We will further discuss about performance and applications of these approaches with our models, particularly when genetic data are spatially explicit and different spatial scales are incorporated into the data.

### 2.4 Individual‐based analyses

We used the principal component analysis (PCA) function (Jongman, Braak, & van Tongeren, 1995) in the *R*‐package *ade4* (Dray & Dufour, 2007) and *pca3d* (Weiner, 2013) for clustering and ordination plotting. Spatial autocorrelation in the principal components of the PCA was tested using Moran’s *I* (Moran, 1950) and Monte Carlo randomization with 1,000 permutations in the *R*‐package *spdep* (Bivand, Pebesma, & Gómez‐Rubio, 2008). With spatially explicit ordination, we used spatial PCA (sPCA) in the *R*‐package *adegenet* (Jombart, Devillard, Dufour, & Pontier, 2008) to detect any correlation between geographic distance and genetic variation. sPCA is considered useful tool to identify fine‐scale spatial patterns of genetic variability when mapping the spatial components of the principal components’ scores. As suggested by Jombart et al. (2008), we modified the default symmetrical Gabriel connectivity graph by inputting the coordinates of our sampling locations. Scores in the first principal component in our sPCA model were then regressed onto the sampling localities using linear least squares. Residual values were interpolated by inverse distance weighting across the sampling region. We used QGIS 2.4 Chugiak (QGIS Development Team 2014) to construct visual representations of the data.

We constructed correlograms (Sokal, 1986) to estimate the presence of genetic relatedness across the spatial range in our AFLP dataset. A correlogram is different from ordination as it is not a global statistical procedure per se (e.g., dividing and estimating relatedness by spatial ranges). Instead, it provides an image of a correlation statistic, here the influence of spatial range on genetic relatedness, i.e., IBD. IBD is expected to occur if dispersal is restricted by distance, for example, decreasing genetic relatedness as a function of geographical distance for the entire sample. IBD does not occur when a feature in the landscape (e.g., a landscape barrier) does not allow or impedes free movement and dispersal. In this study, we employed the *R*‐package *ncf* (Bjornstad, 2013) to construct a Mantel multivariate (cross) correlogram with 1,000 permutations (Bjornstad, Ims, & Lambin, 1999; Mantel, 1967) and discrete distance classes of 1 km increments.

### 2.5 Population‐based analyses

We first estimated the magnitude of genetic variation in our AFLP dataset with *AFLPsurv* (Vekemans, 2002) with a Bayesian model that established nonuniform prior distributions between samples (Zhivotovsky, 1999). For each observed population, which was defined here as the primary (CTN, HBA, LGO, YDN) and secondary (BDP, NCA, TKU) sampling sites, we calculated the expected heterozygosity (*H*_E_), proportion of polymorphic sites, and pairwise *F*_ST_. The significance of the latter was tested with a randomization procedure consisting of 1,000 permutations to create a 95% confidence interval (CI). This procedure tests the null hypothesis of no genetic differentiation among the populations. If the value of *F*_ST_ at the 5% rightmost portion of the CI is less than the observed *F*_ST_, the null hypothesis is rejected. We used the rarefaction function in the *R*‐package *vegan* (Oksanen et al., 2013) to create thresholds for expected private allelic richness in equal sized random subsamples from each population (Kalinowski, 2004) and then recorded the observed private alleles that were greater than the threshold of 5% of the total subsample size.

We used the Bayesian clustering method in GENELAND 4.0.4 (Guillot, Mortier, & Estoup, 2005) with admixture (GENLAND versions <3.3.0 do not have an admixture model) and correlated allele frequencies to identify if distinct genetic clusters were detectable in the samples of Red Junglefowl. Similar to band‐based ordinations, we used both a spatially explicit clustering model (using sampling geographic coordinates and genotypes, similar to sPCA) and a nonspatial model (genotypes only, similar to traditional PCA). Both models were subjected to 2 × 10^6^ Markov chain Monte Carlo (MCMC) iterations, thinning by a factor of 100. A posterior burn‐in of 2,000 iterations was allowed (i.e., 2 × 10^5^ burn‐in out of the total 2 × 10^6^ MCMC iterations). We ran each model 1,000 times on UW‐Madison’s HT Condor computer cluster and computed the average posterior distribution of the modal Δ*K* population. We also constructed a dendrogram of all replicates using average linkage of unweighted pair group method with arithmetic mean (UPGMA). The UPGMA topology reflects the mean posterior probability of common genetic cluster memberships observed from the MCMC estimation of Δ*K*. Importantly it does not depict phylogenetic relationships. A distance metric of 0.00 indicates that two Red Junglefowl were always placed in the same population cluster in 1,000 replicates, whereas a distance of 1.0 indicated that the two Red Junglefowl were never grouped together in any of the replicates. Red Junglefowl that consistently changed their memberships in both global and local clustering models may represent individuals sampled from rare, under‐represented, or cryptic populations.

Apart from the stochastic nature of the MCMC sampling and the high dimensionality of the dataset, biological phenomena will influence how individuals are apportioned into specific population. First is the relative relationship between our sampling scale and the spatial range of the Red Junglefowl, which we assumed is modulated by home‐range demography and possibly the intrinsic quality of the sampled habitats. Increased habitat connectivity may increase dispersal, thereby preventing genetic differentiation, ultimately reducing the resolution of inferred genetic clusters. Second, as our sample design was opportunistic within sampling sites, some Red Junglefowl may be sampled from cryptic linages or populations that are genetically quite different than the average genetic difference of Red Junglefowl in our sampling pool. If individuals meeting these criteria are sampled at low density, assignment into specific populations will be more difficult to achieve, particularly at the global scale of our sampling design. We addressed the global sampling issue by running additional Bayesian clustering, as well as sPCA ordinations, for each individual sampling site. The additional models for the local sampling sites (hereafter *local models*) were parameterized similarly to the models with all samples included (hereafter *global models*). Correlogram models were calculated only at a local scale and had discrete 1 km distance classes up to the maximum sampling distance.

## 3 RESULTS

A total of 212 birds were sampled from the seven field sites (Table 1; Figure 2). Age and sex of the captured birds were determined by phenotypic characteristics including weight, the presence/absence of combs and wattles, plumage, and spur size. Based on these characteristics, we acquired 172 roosters, 23 hens, and 17 juvenile chicks (<3 months old). Within sites, we engaged opportunistic sampling strategies depending on the number of Red Junglefowl territories that we could identify. The average distance between successful captures was 1.12 km (max 26 km, min 0 m of birds in the same flock). Three sites (BDP, NCA, and TKU) had only between five and nine birds sampled due to low abundance of Red Junglefowl living in unsuitable habitats (small natural habitats in coastal lowlands habitats or elevation higher than 600 m). We excluded these sites from our population‐based analyses. Capture rate was roughly only one bird per work day and we found that Red Junglefowl remained highly elusive and vigilant, yet strongly territorial, during their mating season. We attempted to capture equal numbers of male and female Red Junglefowl to evaluate home‐range size and dispersal patterns between sexes. However, we caught significantly more males than females in our study likely because we supplemented our trapline captures with male baiting cocks. Highly territorial Red Junglefowl males responded almost immediately to the presence of these domesticated males, which usually resulted in a successful capture. The use of walk‐in snares with traplines alone had very low capture efficiency (1 bird/0.5 km trapline/day) and required considerably more effort to maintain. With the assistance of baiting cocks, we increased capture efficiency up to four birds per day total but biased our samples toward territorial male Red Junglefowl.

**Table 1:** Genetic diversity of Red Junglefowl in seven field sites based on 398 AFLP loci

Sampling sites	*N*	PLP	*H*_E_ (±*SE*)	PA
BDP	5			
CTN	44	0.445	0.1533 ± 0.0086	16
HBA	56	0.427	0.1492 ± 0.0086	8
LGO	34	0.368	0.1243 ± 0.0083	9
NCA	6			
TKU	9			
YDN	58	0.458	0.1916 ± 0.0089	33

Sample sizes (*N*); proportion of polymorphic markers (PLP); expected heterozygosity (*H*_E_) with standard error (*SE*); private alleles (PA). Genetic diversities were only estimated at study sites with >30 samples.

### 3.1 AFLP fingerprinting

A minimum of two and a maximum of five replicate AFLP fingerprints were generated for each individual bird. After marker binning, the two selective primer pairs yielded 431 replicable polymorphic AFLP loci ranging from 50 to 616 bp, of which 90% (*N* = 389) were polymorphic, that is, had both the presence and absence of marker bands.

### 3.2 Patterns of genetic differentiation

**Figure 3:**
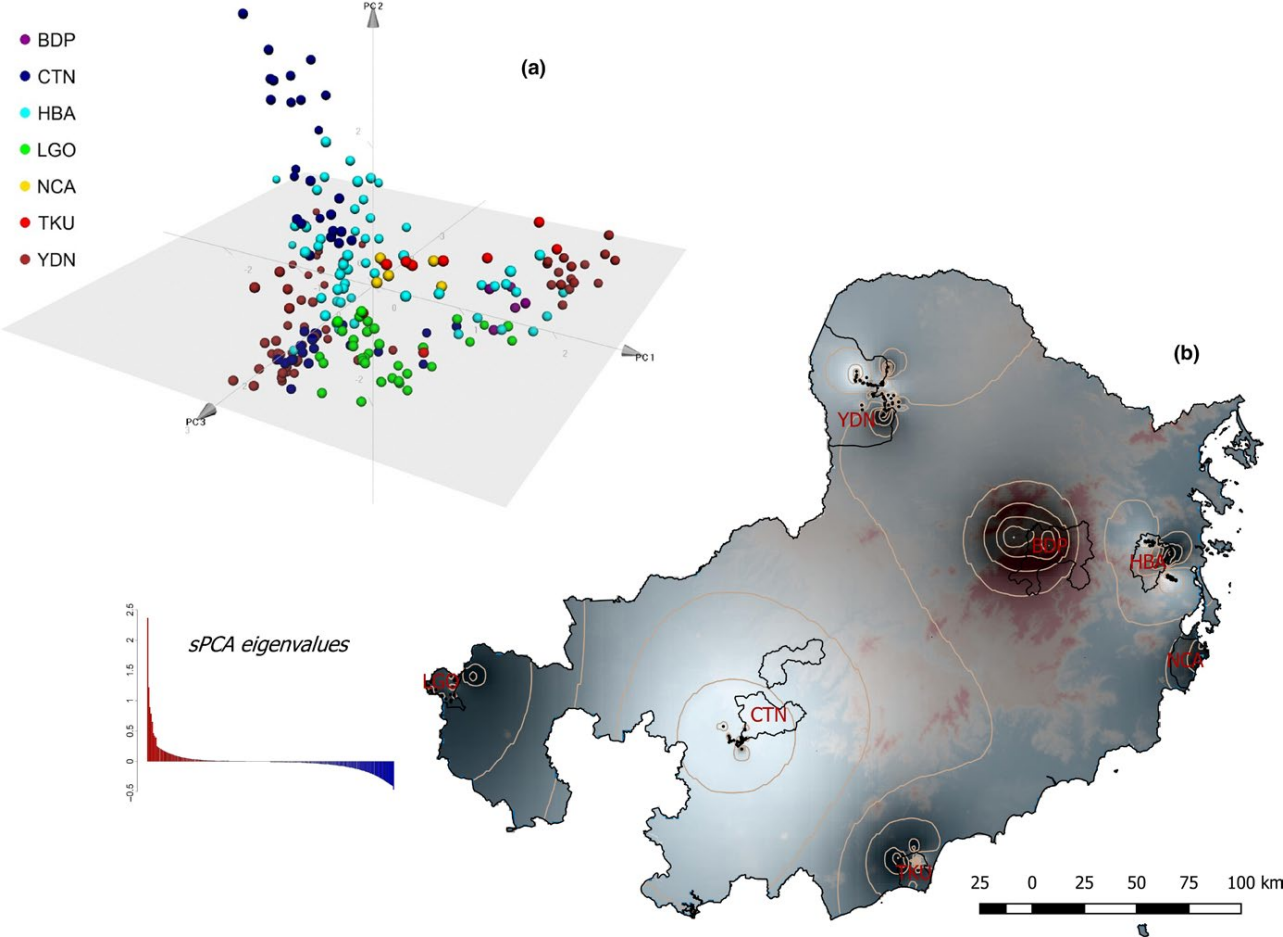
Distance‐based method of genetic variation by principal component analysis (a) and spatial principal component analysis (b). (a) PC: principal component. (b) residual values of regressed principal scores (at global scales) to sampling localities. Dots: Red Junglefowl samples. Contours: component scores for similarity

We observed noticeable patterns of Red Junglefowl in our PCA model (Figure 3a). The first principal component (PC) of our PCA (explaining 10.43% of total variance, Moran’s *I *= 0.49, *p *< .001) separated Red Junglefowl by their sampling locations. Noticeable structure within our two largest and least disturbed sampling sites (lowland tropical forest CTN and highland dry forest YDN) was also evident. The second PC (5.57% of total variance, *I *=* *0.42, *p *< .001) and third PC (4.35% of variance, *I *= 0.42, *p *< .001) supported the observed patterns in PC 1 with CTN and YDN, again, have noticeable within‐site structure. Red Junglefowl sampled in HBA, which is in the northeast foothills of the Annamite Mountain Range, occupied central positions in the PCA plot and then showed some overlap with the Red Junglefowl in CTN and YDN, both of which are further west of the Annamite Mountain Range. This observation implied some low level of genetic exchange among Red Junglefowl across the Annamite (c.f. stepping‐stone model), but it could also be an artifact attributable to both low power and low resolution of PCA model.

Application of the sPCA model at a global scale resulted in high positive eigenvalues and uniformly low negative eigenvalues (Figure 3b). Combined with the overall well‐defined sPCA’s regressed gradient variances (Appendix S1A), this result illustrated monotonic clines of genetic similarities along the east and the west sides of the Annamite Mountain Range landscape. At the local scale, we found evidence of genetic structure within individual sPCA models in the four major sampling sites (CTN, LGO, HBA, and YDN) (Appendix S1A). Both the eigenvalues (not reported here) and residual values of local sPCA scores indicated that Red Junglefowl in lowland habitats south of the Annamite (CTN and LGO) had less within‐group genetic stratification (i.e., fewer genetic clusters represented within each site). Red Junglefowl in the northern Annamite highlands (HBA and YDN) had the opposite spatial pattern, with more genetically distinctive subpopulations identified (higher positive eigenvalues, not reported here) and lower within‐group genetic stratification (dense contours of the sPCA scores).

Spatial autocorrelation analyses of cumulative distance classes indicated that Red Junglefowl exhibited genetic correlation at fine scales and were very site specific (Appendix S1B). HBA (and to a lesser degree, YDN) showed high degrees of genetic relatedness between closely neighboring Red Junglefowl. The magnitude of spatial autocorrelation then declined steadily to a distance of approximately 5 km, beyond which it had negative correlation. The two lowland sites of CTN and LGO showed no autocorrelation at distances similar to HBA and YDN. Overall, the four correlograms revealed a transition to negative values at distances of approximately 5–6 km.

### 3.3 Population structure

Genetic diversity characterized by summary statistics showed a high degree of polymorphism and expected heterozygosity (under HWE; also called Nei’s gene diversity). Each primary population contained private alleles, which were most abundant in YDN (Table 1). The overall *F*_ST_ inclusive of all sampling sites (i.e., the proportion of the total gene diversity occurring within relative to among populations) was 0.1028 (95% CI −0.0106 to 0.0111), a value nearly identical to that calculated with AMOVA (0.0986, *p *< .001) (Excoffier, Smouse, & Quattro, 1992). Pairwise *F*_ST_ among the four primary sites ranged from 0.0267 between HBA and YDN to 0.0935 between CTN and LGO (Table 2). Importantly these estimates do not reflect any subpopulation structure, which would increase the magnitude of among‐population genetic differentiation.

**Table 2:** Pairwise genetic differentiation *F*_ST_ and geographic distances

	BDP	CTN	HBA	LGO	NCA	TKU	YDN
BDP		–	–	–	–	–	–
CTN	169		0.0361	0.0935	–	–	0.0518
HBA	63	220		0.0561	–	–	0.0267
LGO	286	141	346		–	–	0.0602
NCA	98	224	49	357		–	–
TKU	166	100	189	236	170		–
YDN	101	180	158	249	198	227	

*F*_ST_ is above the diagonal (only among four major sampling sites with *>*30 samples) and geographic distance (*d*) in km is below the diagonal. Note that the *F*_ST_ estimates are based on sample‐site groups and do not reflect any subpopulation structure (see text for further details).

**Figure 4:**
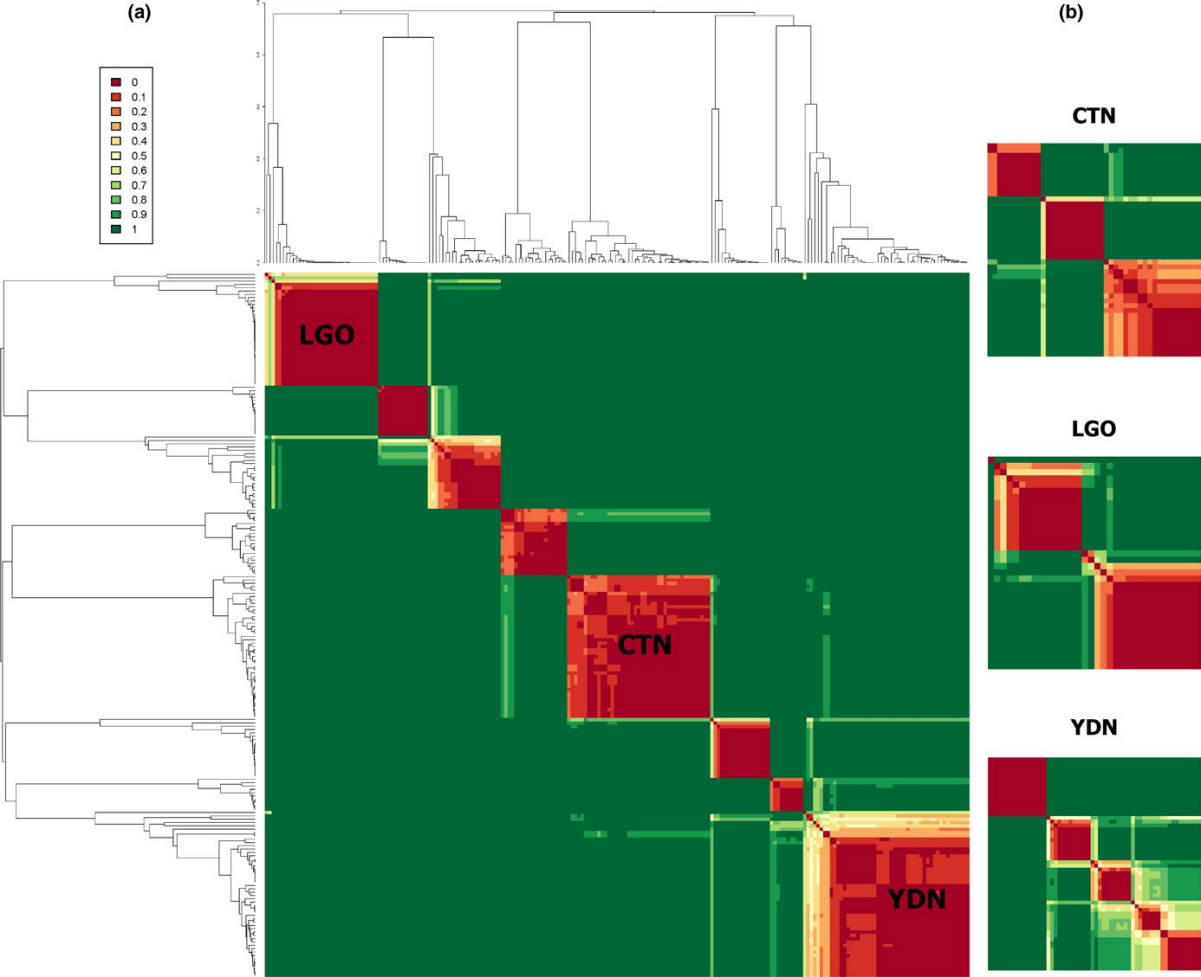
Dendrogram of mean posterior probability of common cluster membership determined from spatial Bayesian clustering and at global scale (a) and local scales (b). UPGMA dendrogram from 1,000 iterations of spatial Bayesian clustering: (a) global scale with three clear clusters (CTN, LGO, and YDN) represent Red Junglefowl, respectively, in the three file sites. Birds in other four sites (BDP, HBA, NCA, and TKU) are clustered in the other unlabeled clusters. (b) local scales: three inserts representing local clustering of LGO, CTN, and YDN

At a global scale, both spatial and nonspatial Bayesian clustering models converged on an estimate of a modal Δ*K* of nine clusters from the seven study sites (Figure 4, results from spatial GENELAND model). Generally, results from the Bayesian clustering method shared similar structure and patterns to results previously obtained with PCA (Figure 3). In both clustering models, Red Junglefowl from each of the sampling geographic sites tended to cluster with birds from the same region. There were some exceptions as a few clusters did include a single individual from another geographic area. This was also evident in the dendrogram depicting the average posterior distribution of individual among the inferred Δ*K* clusters (Figure 4, lighter colored individuals). These individuals probably represent genetically unique forms sampled only once, perhaps at territorial boundaries. However, we cannot confirm this hypothesis without further sampling.

The nine genetic clusters include three (or four) distinct clusters for each of three sites west of the Annamite (CTN, LGO, and YDN—here YDN is larger site and had two subclusters) and five clusters loosely representing HBA Junglefowl and Junglefowl from the other sites (one for the far‐east coastal region HBA‐NCA‐TKU, two northern highland clusters HBA‐BDP and HBA‐YDN, a cluster along the east side of the Annamite HBA‐CTN, and one small cluster with Junglefowl from more than three sites). Although cluster membership in this topographically heterogeneous landscape appears stable (Figure 4), the latter cluster could again be an aggregate of individuals with genotypes sampled at low density. The inferred cluster memberships were almost identical between the spatial and nonspatial model (regression *R* ^2^ = .788, *p *< .001). In both the spatial and nonspatial models, the modal number of populations was Δ*K* = 9 (43% of 1,000 runs) (Appendix S2, results only from spatial models).

We identified additional population stratification at local scales (Figure 4; Appendix S1C–D). Red Junglefowl in CTN were assigned among a modal Δ*K* of three clusters (Appendix S1D) (61% of 1,000, not reported here) instead of two as inferred by the global model (Appendix S1C). This phenomenon may be explained by the clustering algorithm failing to detect sufficient allelic differentiation among the aggregate number of genotypes at the global level. Biologically, the occurrence of three genetic clusters is supported by field conditions in CTN. Here, Red Junglefowl were sampled from two relatively disturbed bamboo forests in the middle of the reserve and a large well protected region connected by northern corridor (Appendix S1D—represented as two regions at the two sides in the map). Similar occurrences were also noted in the other field sites. HBA had one additional cluster (from 4 to 5 in 42% of replicates) at a local scale with the more disturbed central region separated into two different clusters (Appendix S2D). The two topographically flat sites, LGO and YDN, also yielded more population clusters under the local models (Appendix S2D) (from 1 to 4 in 74% of replicates in LGO, and from 2 to 4 in 55% in YDN). However, with LGO and YDN, we did not find a clear link between pattern of cluster assignment and local landscape features or geography patterns and our models mostly assigned Junglefowl sampled next to one another to same clusters. This genetic clustering pattern is possibly a pattern of IBD and limited dispersal where Red Junglefowl demography is more deciding factor than landscape barrier which does not exist at the sites.

As commonly done, calculation of *F*_ST_ (Table 2) was based originally on a per sampling site basis, defining the population as a broad geographical region (i.e., CTN, HBA, LGO, and YDN). At a global scale, the Bayesian clustering methods determined a modal Δ*K* of nine clusters (Figure 4a), and at a local scale, up to 18 additional clusters were inferred (Figure 4b). Estimates of *F*_ST_ using populations defined only by the sampling site likely underestimated the true magnitude of differentiation. In such cases, *F*_ST_ would be downwardly biased when inter‐population heterozygosity varied among the sampled individual populations (Hedrick, 2005), as would be the case in spatially structured populations. When *F*_ST_ was estimated with subpopulations defined by inferred genetic clusters (e.g., the global Δ*K* = 9 and local Δ*K* = 18 clusters), the overall *F*_ST_ increased to 0.1974 for the entire sample, ranging up to 0.3169 between individual sites, with the majority of among‐group comparisons greater than 0.1500. This confirmed the strong population structure of Red Junglefowl in South Central Vietnam as already observed by the ordination and Bayesian methods.

## 4 DISCUSSION

### 4.1 Spatial pattern and population structure

We found large amounts of genetic diversity and strong population genetic structure in wild Red Junglefowl at coarse geographic scales spanning the Annamite Mountain Range of South Central Vietnam. At a local scale, we also found strong evidence of fine‐scale genetic subdivision at distances as low as 5 km. The average sampling distance of 1.12 km in our study appears to be appropriate for detecting genetically divergent groups of Red Junglefowl, especially as we did not detect IBD at distances greater than 5 km. Here, we discuss the importance of genetic structure and the scale of spatial ranges in wild Red Junglefowl, and the implications of these results for genetic management and conservation.

At a broad (regional) scale, the Annamite Mountain Range is likely an impassable barrier limiting long‐distance dispersal of ground‐dwelling organisms, including Red Junglefowl. Although it has been reported that Red Junglefowl occur at elevations up to 1,800 m (Johnsgard, 1999), we rarely observed it above 600 m. Red Junglefowl sampled at higher elevations (e.g., BDP and to a lesser extent HBA) also exhibited substantial genetic differentiation from lowland populations. Although our sample sizes of Red Junglefowl at higher elevations were limited, this result may suggest altitudinal population stratification, a known phenomenon (Stevens, 1992).

In the northwest highland site YDN, we observed even greater genetic differentiation between local Red Junglefowl populations and populations in other lowland sites (CTN, LGO). We also found strong differentiation between sites in the southern lowlands (CTN) and eastern coastal region (NCA, TKU). Biogeographical and climatic phenomena may help explain this result beyond a distance‐only interpretation. South Central Vietnam is considered at the convergence of two biogeographic regions, mostly due to the Annamite Mountain Range: the Annamese Mountains Region consists of subtropical drier monsoon habitats in the northwest uplands (including BDP and YDN) and the Cochinchina Region of moist tropical lowlands in the south and an acrid microclimatic region in the eastern coast (including other five sites—HBA shares some Annamese habitats) (MacKinnon, 1997; Sterling & Hurley, 2005). The inferred genetic clusters of Red Junglefowl in our study were geographically concordant to these regions and confirm the importance of the Annamite to broad‐scale population genetic diversity of this species.

At local scales, the coastal sites of NCA, TKU, and part of HBA consistently form a single cluster even though the sites are separated by approximately 110 km. This contrasts sharply Red Junglefowl sampled in YDN where distinct population clusters are observed (Figure 4) even across short spatial distances of a few kilometers. From an ecological perspective, natural dispersal and movement of Red Junglefowl in the coastal region among HBA, NCA, and TKU is highly unlikely, at least in the current or recent time. There are no existing corridors or landscape connectivity between these sites, and human population densities are extremely high in this region. It would be interesting—and necessary—to further study incongruences between Bayesian clustering and the reality of current landscape connectivity. At this time, we can only presume that historical connectivity, possibly influenced by human‐assisted movement are combined factors that shaped the population structure in the coastal area. Humans have inhabited in coastal areas of South Central Vietnam for well over a 1,000 years and this may influence local Junglefowl populations.

Alternatively, this occurrence may be similar to the “long branch attraction” (LBA) phenomenon, where distantly related lineages are incorrectly inferred to be closely related (Felsenstein, 1978). We were unable to find any reference to LBA with respect to GENELAND and other Bayesian clustering models but note that LBA occurs also in Bayesian phylogenetic studies (Kolaczkowski & Thornton, 2009). We are performing empirical simulations to determine whether the LBA problem may influence clustering models.

Importantly, the combination of broad‐ and fine‐scale spatial analyses in the study suggested distinct characteristics of a classical (Levins) metapopulation structure of Red Junglefowl in South Central Vietnam. Our current evidence suggests that a fragmentation model does not apply. Overall, we observed well‐defined geographic distributions and inferred some evidence of admixture between sites with close geographic proximity, for example, between the southern lowland sites (CTN, LGO), the northern highlands (YDN, BDP, HBA), or between the apparent panmictic coastal sites (HBA, NCA, TKU). Long‐distance genetic similarity was rarely observed, and if so it may have resulted from a LBA‐like phenomenon. A classic stepping‐stone model may also not be suitable as we did not find strong correlation between geographic distances and genetic dissimilarity (IBD) at all of our primary sites. Increased sample numbers and additional study of Red Junglefowl demography may resolve this issue. Although we cannot be certain with the current data, we conclude that these observations at least corroborated a classical metapopulation structure in Red Junglefowl. Moreover, a metapopulation structure resulting from fragmentation of a formerly continuous population or a model of completely subdivided populations is not supported either as we observed clear differentiation between inferred genetic clusters, which generally occurred in contiguous and suitable habitats.

### 4.2 Spatial sampling scales and model performance

The MCMC procedure in any Bayesian clustering method—including the GENELAND models employed here—functions by randomly resampling the data as a basis for its inference. When a regular increase or decrease in correlation between genetic variability and geographic distance occurs (i.e., IBD), it may disrupt the resampling process and generate false‐positives in Bayesian randomization (Meirmans, 2012). With IBD, MCMC models likely fail to explain spatially explicit genetic variation (Frantz, Cellina, Krier, Schley, & Burke, 2009; Schwartz & McKelvey, 2009). There are a few biological and technical procedures to accommodate the presence of IBD, including stratified sampling (as employed in this study) (Storfer et al., 2007) and correlogram analyses to determine whether or not IBD patterns exist. Beyond approximately 5 km, the correlograms did not detected significant IBD (Appendix S1B) and we observed consistent clustering results between our spatial and nonspatial models. We concluded that our Bayesian clustering inferences were robust and reflect accurately the information contained in the AFLP data.

Balkenhol (2009) reviewed different Bayesian model parameters and the importance of combining different methods in clustering procedures for population and landscape genetics studies. In this present study, we emphasized the application of Bayesian MCMC replicates. With support of a high‐performance supercomputing system, we could compute large runs of Bayesian iterations (1,000) with sufficient burn‐in and chain lengths. Many similar studies invoke between 5 and 10 replicates, a number probably too small to sample such high‐dimensional space. The large number of replicates allowed us to construct average posterior densities of individual population membership (Appendix S2) and served to add an additional interpretive dimension. This also allowed us to identify individuals that may have originated from cryptic or unsampled populations. From statistical point of view, increased resampling of data from high‐dimensional MCMC should improve the interpretive reliability of the underlying genetic characteristics in our dataset (Guillot, 2008). On the other hand, deterministic and computationally simpler individual‐based methods such as PCA and sPCA models can also provide intuitive information about genetic variation from reasonably sized datasets exhibiting strong population structure. Ordination methods also helped to provide an analytical framework from which we accordingly developed the more sophisticated analyses.

Another important point with spatially explicit Bayesian clustering is that a caveat exists in terms of model performance and spatial sampling scales. In our work, we found that random sampling (e.g., complete spatial randomness, CSR) does not hold at very fine scales, as most living organisms, including Red Junglefowl, are genetically related when they are sampled at close enough distances (Guillot et al., 2009). This represents trade‐offs between global and local scales in model performance: Global models (with all data points included) generally met the implicit requirement of CSR but provide low resolution when identifying areas of genetic boundaries and/or transitions. Local models, on the other hand, can be useful in identifying genetic structure in clines and transition areas, for example, between less disturbed forests and areas with more human disturbances. In either case, interpretations should be treated with caution and full understanding of the model(s) used.

### 4.3 Introgression and conservation of Red Junglefowl

The effects of genetic introgression are critically important when interpreting genetic studies of wild Red Junglefowl. This issue requires consideration from different perspectives, including at least its ecology and behavior, the source and manner in which introgression occurs, and how introgression is detected. Defining wild‐type Red Junglefowl in their natural habitats should be a priority but is not necessarily simple. As mentioned in the Introduction, the results from several previous studies with Red Junglefowl should be interpreted with caution as samples came from captive colonies, which may not represent accurately wild‐type characteristics, for example, behaviors and phenotypes (Brisbin, 1995; Brisbin & Peterson, 2007; Brisbin et al., 2002; Jaap & Hollander, 1954; Peterson & Brisbin, 1998) each of which may have been affected by genetic introgression.

Evaluations of genetic introgression and establishment of a conservation profile of wild Red Junglefowl in South Central Vietnam may depend on specific evolutionary processes and genetic markers. In a previous analysis with the same Red Junglefowl individuals used in this study (*n *=* *199 excluding birds in BDP, NCA, TKU, and including some additional samples from CTN and LGO), we assayed MHC B‐locus haplotype variation. We found 310 of 398 (78%) unique Red Junglefowl haplotypes. Of the 310 unique haplotypes found, none were identified in any commercial (*n *=* *1,359) or local heritage chickens (*n *=* *32) (Nguyen‐Phuc et al., 2016). The sample of heritage chickens also included “ga tre,” a species known to be crossed artificially with wild male Red Junglefowl.

In the current work, we compared our population‐level results with other studies (e.g., Akaboot et al., 2012; Berthouly et al., 2010; Granevitze et al., 2007; Liu et al., 2006; Miao et al., 2013; Nishibori et al., 2005; Okumura et al., 2006; Peterson & Brisbin, 1998) that also addressed genetic exchange between feral or free‐ranging domestic chickens and so‐called wild Red Junglefowl. Advantageously, these studies used Red Junglefowl also obtained from South and Southeast Asia. Among them, Okumura et al. (2006) was the only study that reported genetic variability in wild Red Junglefowl at the population level. The study indicated that “Red Junglefowl (*n *=* *28) were collected in Vietnam, Laos, and Indonesia” (Okumura et al., 2006, p. 189). Without additional information, this statement might [erroneously] imply that the birds were wild type and therefore representative of local Red Junglefowl populations. The degree of *F*_ST_ between the sampled Red Junglefowl and local domestic chicken breeds in Indonesia, Laos, Myanmar, and Thailand was very low, ranging from 0.0100 to 0.0081. The magnitude of *F*_ST_ increased with commercial poultry breeds, ranging from 0.0824 with broilers and 0.1316 with layers.

Although the two studies differed in terms of the genetic marker used (AFLP vs. gene‐specific) and the geographic scale sampled (restricted range vs. multi‐national), the amount of genetic differentiation observed among wild Red Junglefowl populations in South Central Vietnam (this study) was much greater than in the Okumura et al. study. Indeed, the low levels of *F*_ST_ they observed with local domestic breeds suggest long‐term introgression of domestic alleles among the entire sample of Red Junglefowl. The fact that *F*_ST_ increased considerably with commercial breeds, which are generally housed and separated from contact with other chickens, is consistent with this interpretation.

Adding adaptive analyses to discussions of wild Red Junglefowl strengthen the appeal of preserving wild Red Junglefowl and other heritage breeds of poultry. For example, conservation of genetic diversity in agriculturally important species is also required for sustainable agriculture. Climate change, newly emergent diseases, increased pressure on land and water resources, and shifting market demands require that [domestic] animal genetic resources are also conserved and used sustainably. Wild Red Junglefowl and heritage poultry breeds certainly experience adaptive selection, acquiring traits allowing them to adapt to differing local environments and cultural conditions, which are unlike any encountered in modern agriculture. The unusually high variation of MHC B‐locus in Red Junglefowl reflects a substantial evolutionary solution to pathogen resistance, a matter of great concern in commercial poultry operations. Similar to the biological conservation of wild species, the potential to augment commercial poultry genetic management is substantial and both wild Junglefowl and heritage poultry breeds should be considered an invaluable genetic reservoir well worth protecting.

## CONFLICT OF INTEREST

None declared.

## AUTHOR CONTRIBUTION

HNP and MEB conceived the ideas and wrote the manuscript; HNP conducted the field work with assistance from MEB; HNP and MEB analyzed the data. All authors gave final approval for publication.
