# Supplementary figures and images for: Genetic structure in Red Junglefowl (Gallus gallus) populations: Strong spatial patterns in the wild ancestors of domestic chickens in a core distribution range
Source: Ecol Evol. 2018 Jun 11;8(13):6575–88. doi: 10.1002/ece3.4139 (PMC6053552; doi:10.1002/ece3.4139)

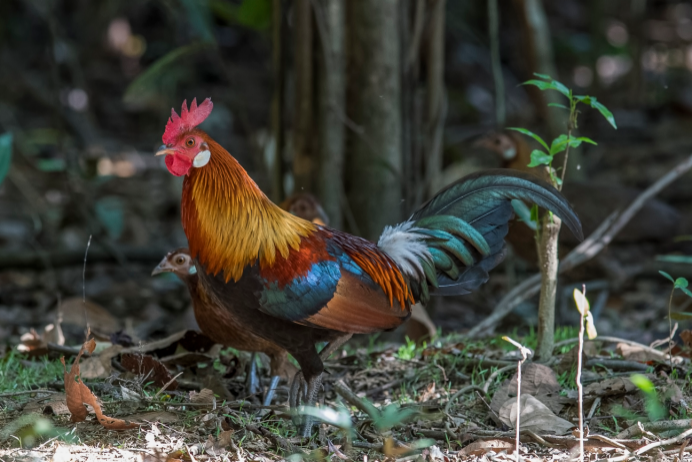

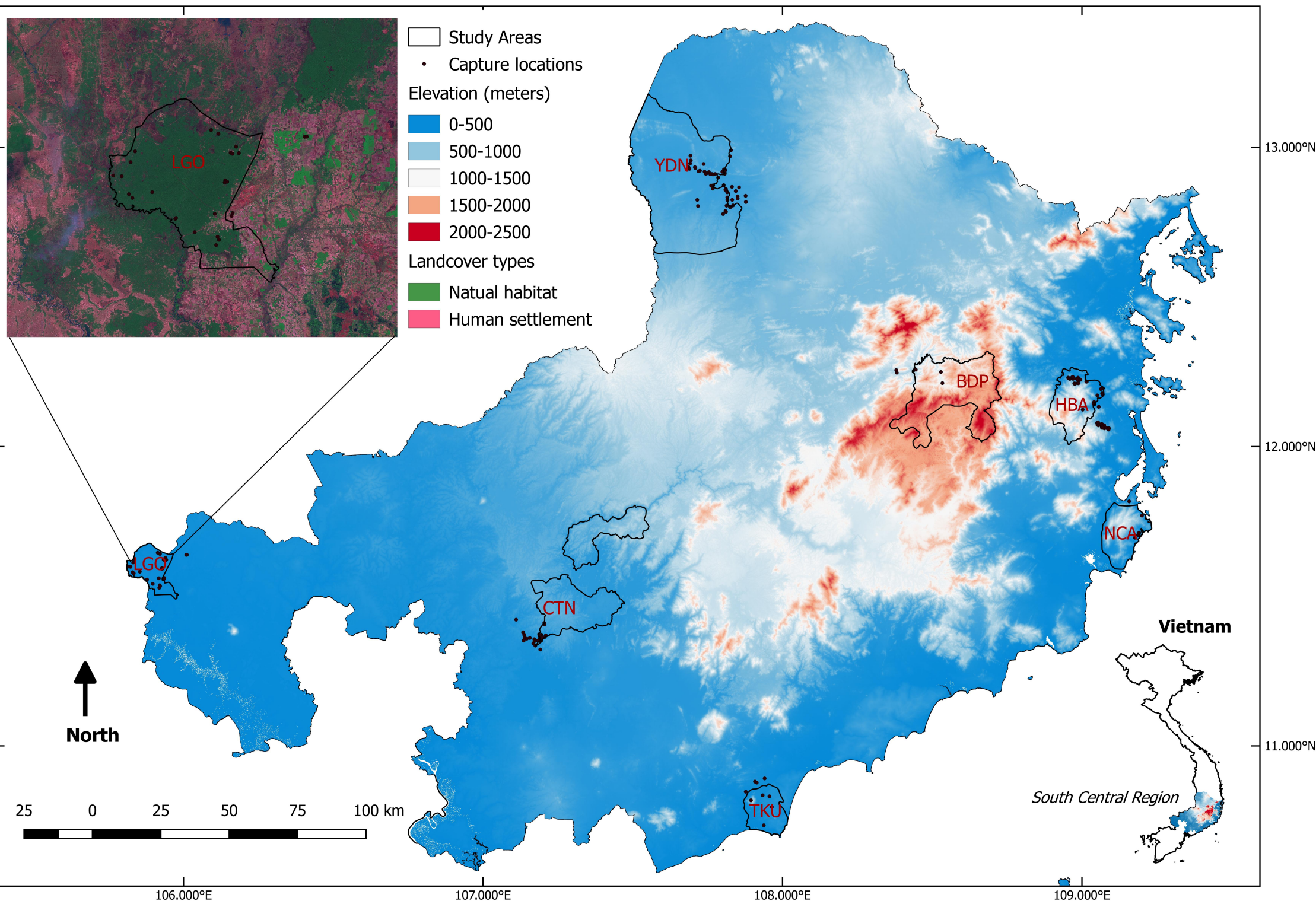

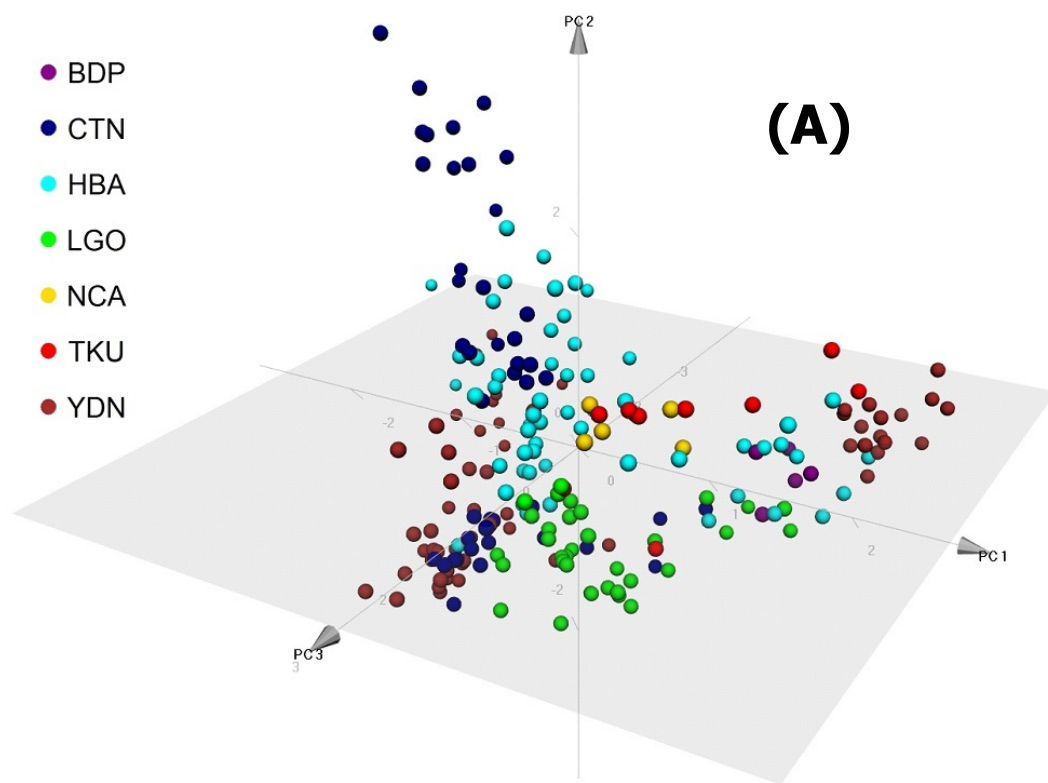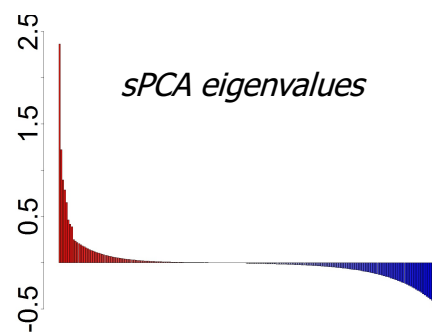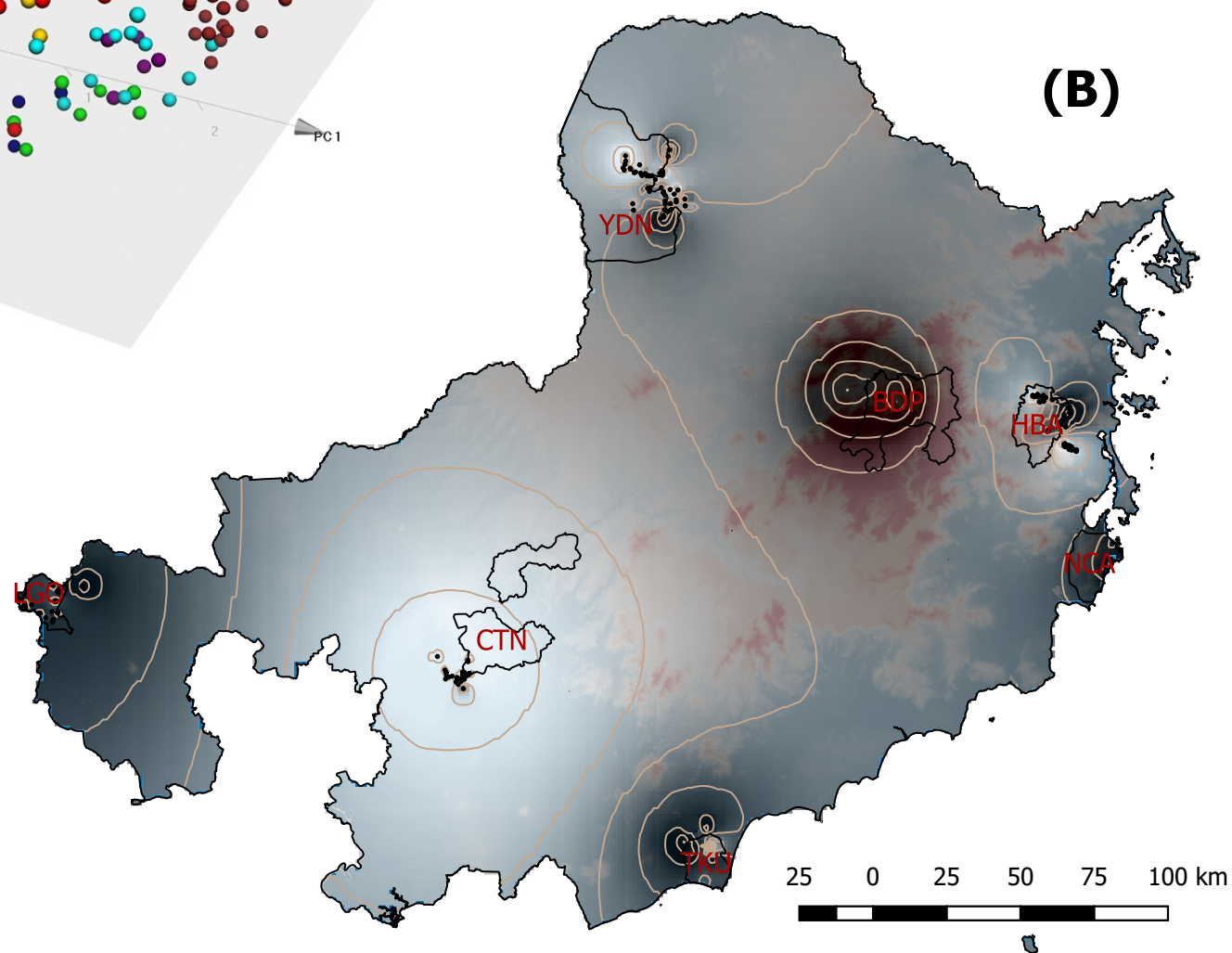

**(A)**

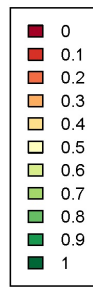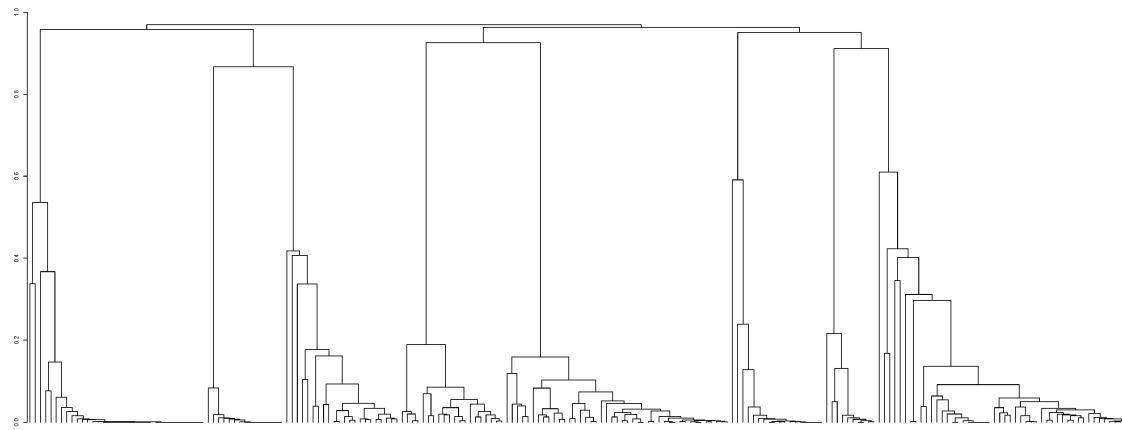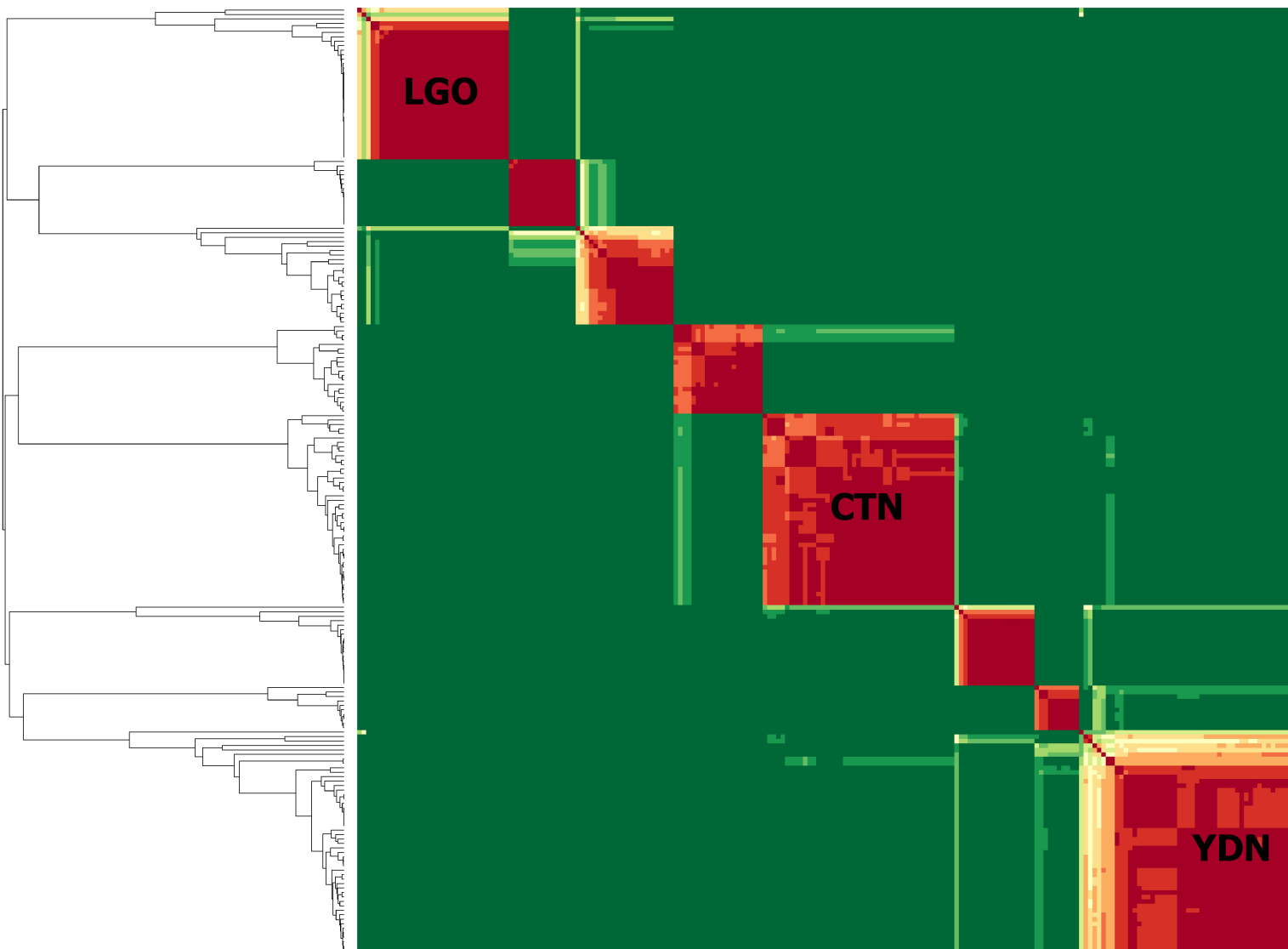

**(B)**

**CTN**

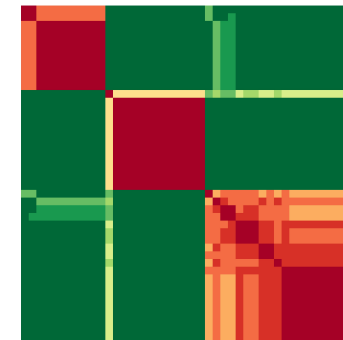

**LGO**

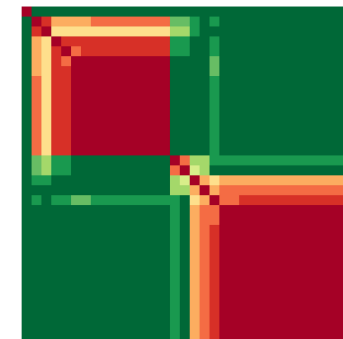

**YDN**

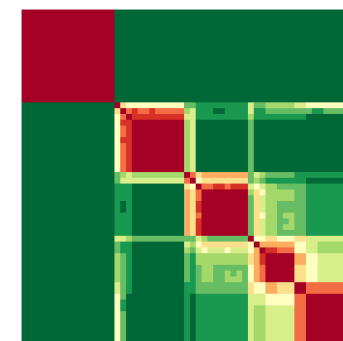

**CTN****LGO****HBA****YDN****(A)**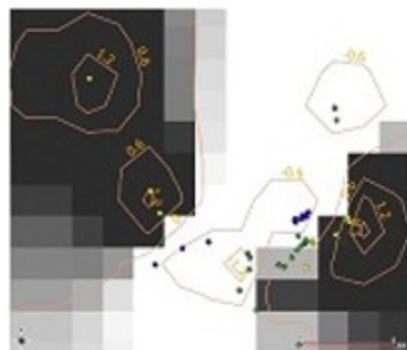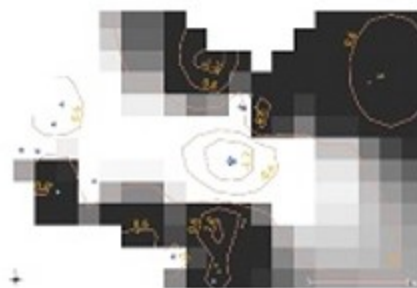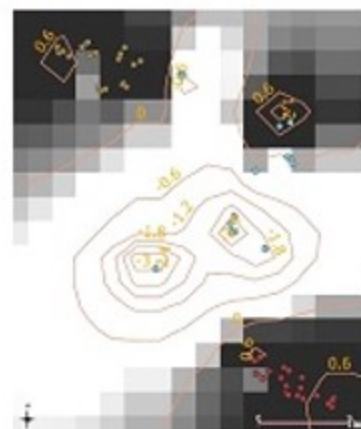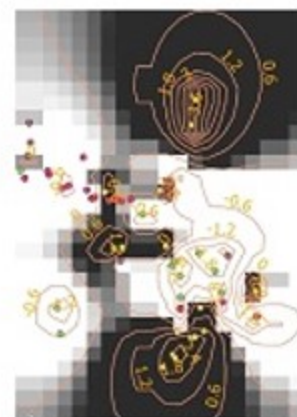**(B)**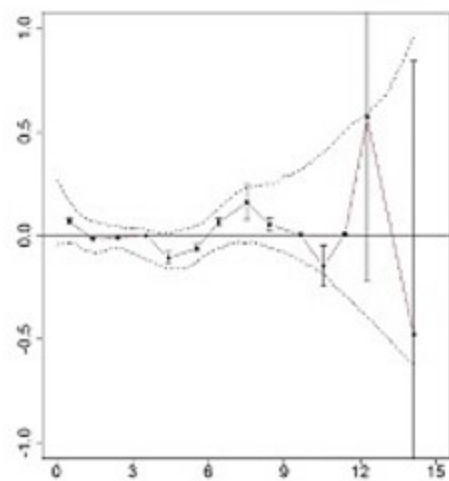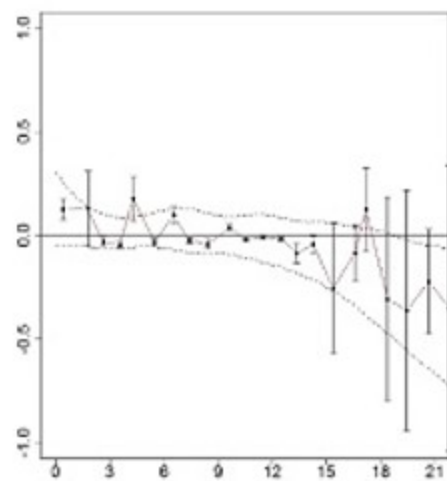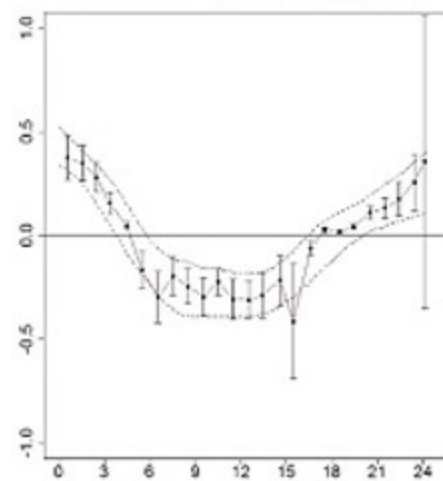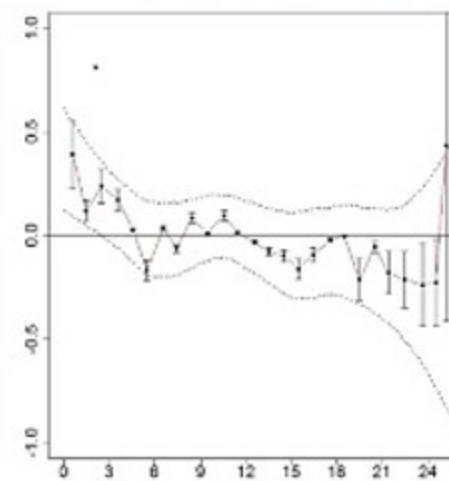**(C)**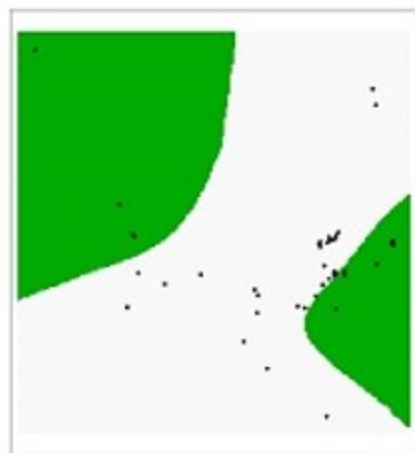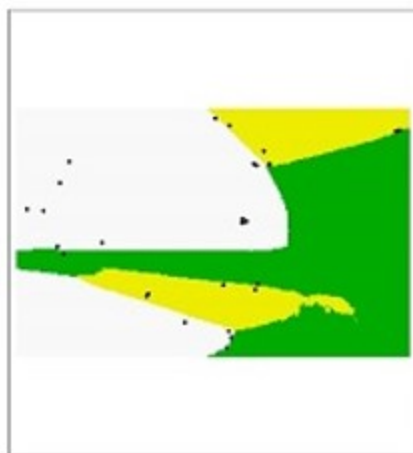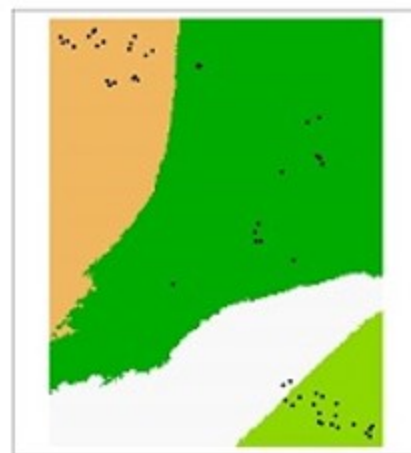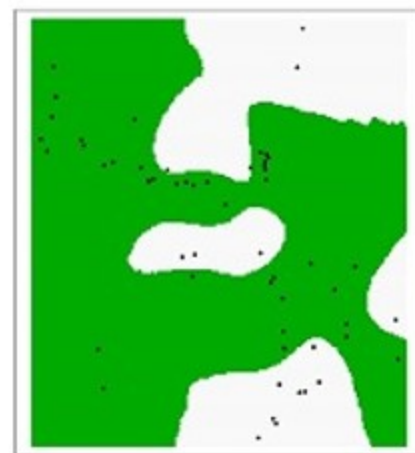**(D)**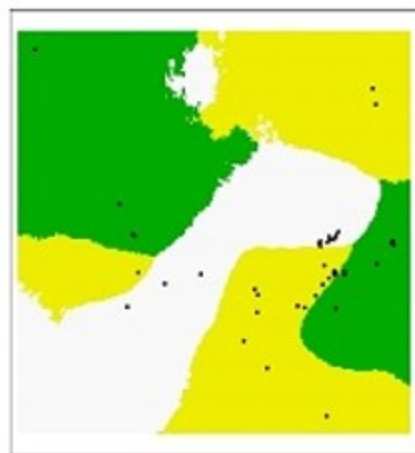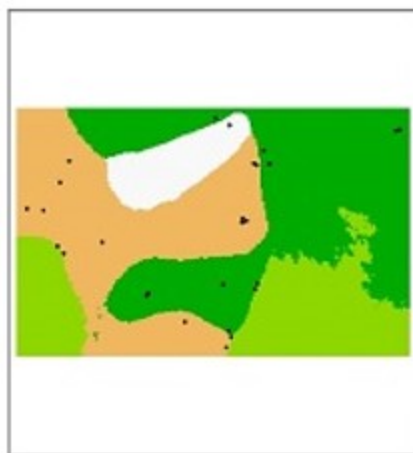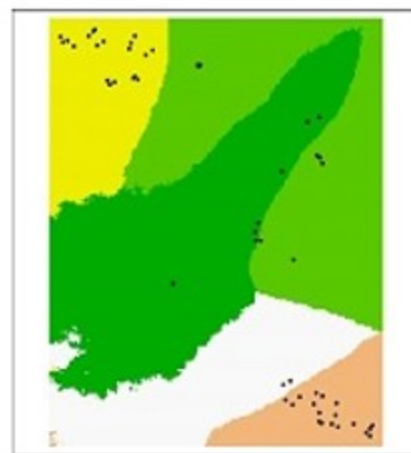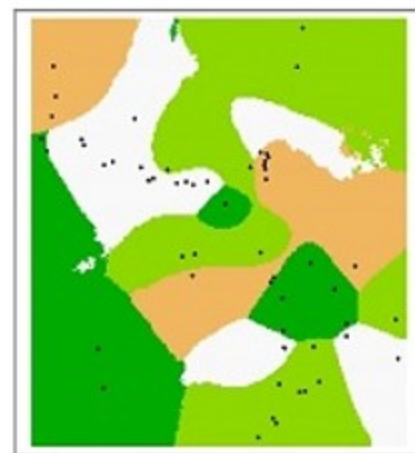

(A)

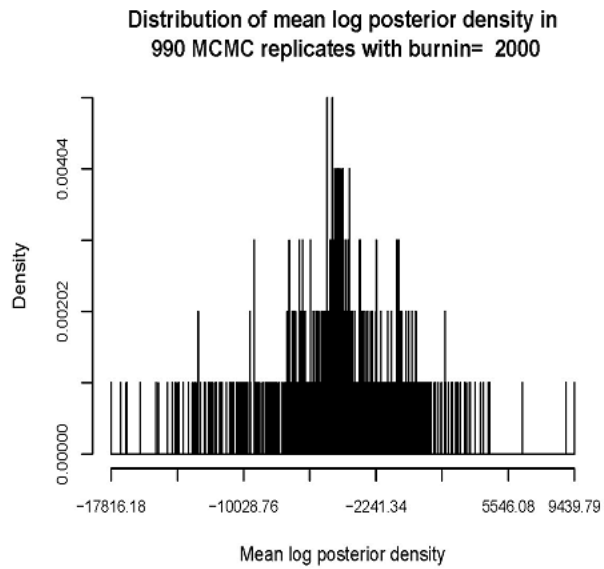

(B)

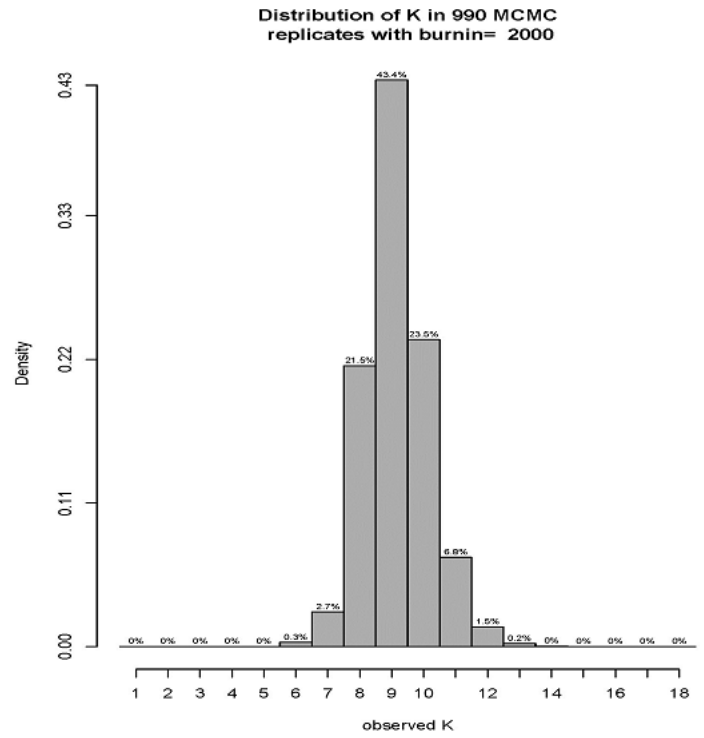

Supplement: Supplementary file 1 [file ECE3-8-6575-s001.pdf]
