# Supplementary material for: Genetic structure in Red Junglefowl (Gallus gallus) populations: Strong spatial patterns in the wild ancestors of domestic chickens in a core distribution range
Source: Ecol Evol. 2018 Jun 11;8(13):6575–88. doi: 10.1002/ece3.4139 (PMC6053552; doi:10.1002/ece3.4139)
Supplement: Supplementary file 2 [file ECE3-8-6575-s002.docx]

**Supplementary Appendix - S1: Spatial population structure.**

(A) sPCA: residual values of regressed principal scores (at local scales) to sampling localities. Dots: Red Junglefowl samples. Contours: component scores for similarity.

(B) Correlogram: *y*-axis: correlations; *x*-axis: cumulative distance classes; error bars: 95% confidence bootstrapped; dashed lines: confidence intervals of 1,000 permutations around the null hypothesis of a random distribution.

(C) Global model of Bayesian clustering and (D) Local model of Bayesian clustering: Red Junglefowl samples, color regions are the posterior probability spatial clusters; plots represent only one run (out of 1,000) that has highest log posterior density.

**Supplementary Appendix - S2: (A) mean log posterior density, (B) distribution of *K* populations of global model of Bayesian clustering.**

Only global run (spatial mode) reported. From 2 *x* 10^6^ Markov chain Monte Carlo (MCMC) and 1,000 iterations, thinning by a factor of 100 and burn-in of 2,000 iterations was allowed.
